# Parathyroid Hormone-Related Protein (PTHrP) Accelerates Soluble RANKL Signals for Downregulation of Osteogenesis of Bone Mesenchymal Stem Cells

**DOI:** 10.3390/jcm8060836

**Published:** 2019-06-12

**Authors:** Jeevithan Elango, Saeed Ur Rahman, Yves Henrotin, José Eduardo Maté Sánchez de Val, Bin Bao, Shujun Wang, Bailin Li, Wenhui Wu

**Affiliations:** 1 Department of Marine Bio-Pharmacology, College of Food Science and Technology, Shanghai Ocean University, Shanghai 201306, China; srijeevithan@gmail.com (J.E.); bbao@shou.edu.cn (B.B.); blli@shou.edu.cn (B.L.); 2 Interdisciplinary Research Centre in Biomedical Materials (IRCBM), COMSATS University Islamabad, Lahore Campus, Punjab 54000, Pakistan; saeedbio80@gmail.com; 3 Bone and Cartilage Research Unit, Arthropôle Liège, University of Liège, CHU Sart-Tilman 4000, Liege, Belgium; yhenrotin@ulg.ac.be; 4 Department of Biomaterials Engineering, Universidad Católica San Antonio de Murcia, 30107 Guadalupe, Murcia, Spain; jemate@ucam.edu; 5 Co-Innovation Center of Jiangsu Marine Bio-industry Technology, Huaihai Institute of Technology, Lianyungang 222005, China; shujunwang86@163.com; 6 National R&D Branch Center for Freshwater Aquatic Products Processing Technology (Shanghai), Shanghai 201306, China

**Keywords:** soluble RANKL, mesenchymal stem cells, PTHrP, osteoblastogenesis, ANK, T-lymphocytes

## Abstract

A recent study reported the expression of receptor activator of nuclear factor-κB (RANK) in mesenchymal stem cells (MSCs) surface that negatively regulates osteogenesis of MSCs. Empirical evidence from the previous study confirmed the role of parathyroid hormone-related protein (PTHrP) in osteoblastogenesis. However, it is necessary to understand the paracrine role of PTHrP and RANKL for osteogenesis in order to explore the hidden secrets in bone biology. Considering the above concept, paracrine cues of soluble-receptor activator of nuclear factor-κB ligand (sRANKL) and PTHrP in osteogenic differentiation of MSCs were investigated. Our results confirmed that sRANKL increased the expression of surface-RANK in MSCs at the earlier stage of osteogenesis, which was downregulated later in differentiated MSCs. In contrast, RANKL expression was low at the earlier stage of MSCs proliferation and high at the differentiation stage of MSCs, which may play a fundamental role in osteoclast formation. sRANKL downregulated osteogenesis of MSCs by decreasing progressive ankylosis (ANK) protein expression while PTHrP upregulated the osteogenic exploitive effect of sRANKL. Interestingly, when they were co-cultured with MSCs, T-lymphocytes expressed high membrane-RANKL levels that contribute to osteogenesis inhibition during MSC differentiation. Thus, our results disclose that sRANKL treatment downregulates osteogenesis of MSCs by increasing RANK expression at the earlier stage of differentiation and by inhibiting ANK. Further, we demonstrated that PTHrP accelerated the downregulating osteogenic effect of sRANKL.

## 1. Introduction

Receptor activator of nuclear factor-κB ligand (RANKL) is a major tumor necrosis factor-related cytokine expressed by a wide range of organs. RANKL is mainly expressed in spleen, bone, lymph nodes, lungs, mammary gland, heart, thymus, brain, skeletal muscle, kidneys, and skin during the entire mouse development [1,2,3]. It is abundantly articulated in stromal, mesenchymal, immune, chondrocytes, osteoblasts, and spleen cells [4]. Our recent research and other previous studies confirmed that the RANKL is secreted by several osteogenic cells such as mesenchymal bone marrow stem cells, pre-osteoblasts, osteoblast lineage cells, mature osteoblasts, and osteocytes and the major source of RANKL is mature osteoblasts that regulate bone marrow macrophage-derived osteoclastogenesis [1,5,6,7,8]. Mice preclinical studies have found that RANKL in the mammary gland is necessary for lactational hyperplasia during milk production. RANKL is also expressed by tumor cells as a mitogen to regulate cell proliferation [9]. The expression of RANKL depends on paracrine and juxtacrine cues of several hormones and cytokines such as a parathyroid hormone, prostaglandins E2, glucocorticoids, vitamin D3, IL-1, -4, -6, -11, -17 and TNFα [4,10,11,12,13]. Understanding the coupling mechanism of RANKL/osteoprotegerin (OPG)/RANK pathways exemplifies the serendipity of bone forming cells in homeostasis.

Parathyroid hormone-related peptide (PTHrP) is secreted by the parathyroid gland, a major endocrine gland. However, PTHrP is sporadically secreted by some malignant cells. In fact, PTHrP was the first peptide hormone isolated as a hypercalcemic factor contributed in humoral high blood calcium level during malignancy [14,15]. PTHrP actually determines the structural mineralization and the rate of chondrogenesis during bone development [16]. At first, it was believed to be a bone demineralization agent as high osteoclast activity in malignant patients. Later, studies also proved the osteogenic potential of PTHrP due to increased bone mass in PTHrP administered rodents, rats and humans [16,17,18,19]. In contrast, Zhang et al. [20] reported that PTHrP downregulated the proliferation of primary chondrocytes isolated from wild mice by suppressing the transcription factors (both Runx2 and Runx3 protein levels).

Two types of RANKL such as soluble RANKL (sRANKL) and membrane RANKL (mRANKL) are circulated in biological systems and play their role. The main difference is that mRANKL presents in the cellular membrane in bound form and acts through cell–cell contact, whereas sRANKL, cleaved from cell membrane by disintegrin metalloproteinase present in the extracellular environment (circulation). Chen et al. [21] recently identified the expression of RANK in mesenchymal stem cells (MSCs) during earlier stage of osteogenesis and further they disclosed that the RANKL downregulates the osteogenesis of MSCs through upregulating β-catenin degradation and p65 phosphorylation. However, the role of RANKL in the presence of PTHrP for osteogenesis of MSCs remains unclear. Therefore, in this study, MSCs isolated from mouse compact bone were differentiated with osteogenic medium along with sRANKL and PTHrP and the effect of PTHrP on sRANKL function towards osteogenesis of MSCs was observed. Further, MSCs were co-cultured with T-lymphocytes, a major source of RANKL and the downregulating efficiency in osteogenesis of MSCs was investigated.

## 2. Methods

### 2.1. Isolation of Mesenchymal Stem Cells

Mesenchymal stem cells from compact bone of 2-week old BALB/c mouse were isolated as per the previous method [22] with slight modification. In brief, the mouse bones (femur and tibiae) were separated from adherent muscles and flushed with complete culture medium (alpha minimum essential medium (α-MEM)–10% fetal bovine serum (FBS)) to remove bone marrow. Then, the compact bones were fragmented into small pieces (~1–5 mm^3^) using sterile scissors and transferred into a 50 mL falcon tube containing 5 mL of digestion medium (α-MEM and 1 mg/mL collagenase type I). The bone pieces were digested for 90 min at 37 °C under agitation (200 rpm). The digestion medium was changed every 30 min. Then the digested bone pieces were seeded into 90 cm^2^ cell culture dishes with 10 mL alpha-MEM–10% FBS medium for 10 days (Figure S1). Upon confluence, the cells were trypsinized using 0.25% trypsin- Ethylenediaminetetraacetic acid (EDTA) solution and split into three cell culture dishes. The cells passaged up to 10 were used for the experiment based on the previous report [22].

### 2.2. Cell Proliferation

MSCs (1 × 10^4^) were seeded in a 24-well culture plate (Costar) along with different concentrations of recombinant sRANKL (R&D Industries, Minneapolis, MN, USA) (10, 50 and 100 ng/mL) and PTHrP (GL Biochem Ltd., Shanghai, China) (10 and 50 nM/mL) [21]. The total cell count was done at different time intervals such as 1, 3 and 7 days using a cell counter (Invitrogen, Countess II Automated Cell Counter, ThermoFisher Scientific, Shanghai, China).

### 2.3. mRNA Expression

MSCs were seeded (1 × 10^5^ cells/well) in 6-well microtiter plates (Costar, Shanghai, China) along with different concentrations of sRANKL and PTHrP: sRANKL50 (50 ng/mL), PTHrP50 (50 nM/mL), sRANKL10–PTHrP10 (10 ng/mL + 10 nM/mL), sRANKL50–PTHrP50 (50 ng/mL + 50 nM), respectively. After 7 day, the cells were harvested for RNA extraction using the TriZol method and complementary DNA synthesized using a cDNA synthesis kit [5].

GAPDH: F-TGA AGC AGG CAT CTG AGG G; R-CGA AGG TGG AAG AGT GGG AG

RANKL: F-CCT GAG GCC CAG CCA TTT; R-CTT GGC CCA GCC TCG AT

RANK: F-GCC CAG TCT CAT CGT TCT GC; R-TAG CTG TCA GCG CTT TCC CT

### 2.4. Expression of Membrane RANK Using Confocal Laser Scanning Microscopy

For immunocytochemistry, cells were grown in the confocal disc (Cat No. 150682, Thermo Fisher Scientific, Shanghai, China) with sRANKL, fixed with 4% paraformaldehyde (PFA) for 15 min and permeabilized with 0.1% Triton X-100 for 15 min at room temperature (RT). Then, the cells were incubated with primary antibody (anti-RANK) overnight and DyLight 594-conjugated secondary antibody (goat anti-rabbit IgG H and L). In another experimental set-up, sRANKL treated cells were stained with fluorescein isothiocyanate (FITC) and 4′,6-diamidino-2-phenylindole (DAPI). Images were captured using a confocal laser scanning microscope (Leica TCS SP8, Leica Microsystems CMS GmbH, Wetzlar, Germany).

### 2.5. Immunophenotyping Characterization of MSCs

The cell surface marker profiles were identified using flow cytometric analysis. Briefly, the cells were collected by trypsin digestion and the cell suspension with a density of 1 × 10^5^ cells/mL was incubated with fluorescein isothicyanate (FITC)-conjugated anti-mouse HLA-DR, CD90 or phycoerythrin (PE)-conjugated anti-mouse CD40, CD45 and CD105 antibody (BD Bioscience, Shanghai, China) in dark at 4 °C for 30 min. The cell suspensions were analyzed using a FACSCalibur flow cytometer (BD Biosciences).

### 2.6. Tri-Lineage Differentiation Potential of MSCs

Tri-lineage differentiation of MSCs was followed as per the previous reports [23,24]. To induce adipogenic differentiation, the cells were cultured in mesenchymal stem cell-adipogenic differentiation medium (ScienceCell, Shanghai, China, Cat No.7541) containing adipogenic differentiation supplement (ScienceCell, Cat. No. 7542) for 21 days. To induce chondrogenic differentiation, the cells were cultured in mesenchymal stem cell-chondrogenic differentiation medium (ScienceCell, Cat No. 7551) containing chondrogenic differentiation supplement (ScienceCell, Cat No.7582) for 21 days. To induce osteogenic differentiation, the cells were cultured in mesenchymal stem cell-osteogenic differentiation medium (ScienceCell, Cat No.7531-b) containing osteogenic differentiation supplement (ScienceCell, Cat No.7532) for 21 days. After induction, the cells were fixed with 4% paraformaldehyde (PFA). The adipogenic, chondrogenic and osteogenic potential of MSCs were confirmed by oil red O, safranin O and Alizarin red staining, respectively.

### 2.7. Effect of sRANKL and PTHrP on Osteogenic Differentiation

For osteogenic differentiation, MSCs were grown in osteoblast condition medium (ScienceCell, Shanghai, China) with the addition of osteoblast growth supplement (ObGS) (ScienceCell) composed of 100 nM dexamethasone, 10 mM beta-glycerolphosphate, and 0.05 mM 2-phosphate-ascorbic acid for 21 days along with sRANKL and PTHrP. The level of mRNA expression (osteocalcin and collagen) was determined as previously discussed.

Collagen I: F-GCG AAG GCA ACA GTC GCT and R-CTT GGT GGT TTT GTA TTC GAT GAC

Osteocalcin (OC): F-CTC ACA GAT GCC AAG CCC and R-CCA AGG TAG CGC CGG AGT CT

### 2.8. The Level of Secretome

The level of secretome before and after osteogenesis in the presence or absence of RANKL and PTHrP was measured using ELISA. Briefly, the cells were treated with RANKL and PTHrP as mentioned before in osteogenic medium. The osteoprotegerin (OPG) and prostaglandin E2 (PGE2) levels were measured according to the manufacturer’s instruction (Sangon biotech Co., Ltd, Shanghai, China).

### 2.9. Cellular Alkaline Phosphatase

The level of cellular alkaline phosphatase (ALP) was measured as per the previous protocol. Cells were treated with sRANKL and PTHrP as discussed earlier and harvested with lysis buffer (10 mM Tris buffer, pH 7.4) and treated with ALP substrate, p-nitrophenyl phosphate (Sigma-Aldrich, Shanghai, China) and read at 410 nm using a plate reader (Bio-Rad Model 550, Shanghai, China). The same volume of sample was used to determine protein content using bicinchoninic acid (BCA) as per the manufacturer’s instruction (Pierce, Missouri, IL, USA). ALP activity was expressed as nmol/min/mg protein.

### 2.10. Cellular Mineral Levels

MSCs were seeded at a density of 1 × 10^4^ cells/well in microtiter 48-well plates and treated with sRANKL and PTHrP for 21 days. The mineralization level was confirmed by observing calcium and phosphate deposition in cultured cells using Alizarin red and silver nitrate staining, respectively [25]. The percentage of mineral staining in bone cells was quantified using ImageJ software (Version 1.52n, https://imagej.nih.gov/ij/).

### 2.11. Western Blot

Total cellular proteins were isolated, quantified and separated using 10% SDS-PAGE and transferred to polyvinylidene fluoride (PVDF) nitrocellulose membranes (Invitrogen) using the iblot-2 dry blotting system (Invitrogen). Protein transferred membranes blocked with 5% bovine serum albumin-phosphate buffered saline with tween 20 (BSA-PBST) were incubated with primary antibodies (Abcam, Shanghai, China) such as anti-GAPDH, anti-RANKL, anti-RANK, anti-Col_1_α_2_, anti-osteocalcin and anti-progressive ankylosis protein (anti-ANK) overnight at 4 °C. Then the membranes were incubated with secondary goat anti-rabbit IgG-HRP for 1 h at 37 °C and exposed to the enhanced chemiluminescent reagent. Images were captured with a Universal Hood II Gel Doc System (Bio-Rad, Rochester, NY, USA).

### 2.12. Role of T-Cells in Osteogenesis

To understand the actual paracrine cues of T-cells, MoLT-4 cells were co-cultured with MSCs with osteogenic supplemented medium without sRANKL treatment. Briefly, MoLT-4 cells with a density of 1 × 10^5^ cells/mL were seeded on top of previously seeded MSCs (1 × 10^4^) monolayer. The cells were cultured for 21 days with osteogenic growth supplement as mentioned earlier. The MSCs without MoLT-4 cells served as control. The osteogenic properties of MSCs were determined by mineral staining.

### 2.13. Statistics

The average mean values and standard error of the mean were calculated from three determinations and statistical significance was determined using one-way ANOVA. Individual differences between mean values were assessed using Duncan’s multiple range tests. The results were also statistically interpreted using SPSS 18.0 (SPSS 18.0 for Windows, SPSS Inc, Chicago, IL, USA) to determine the least significant differences (LSD) at *p* < 0.05.

## 3. Results

### 3.1. Immunophenotyping Characterization of MSCs

**Figure 1:**
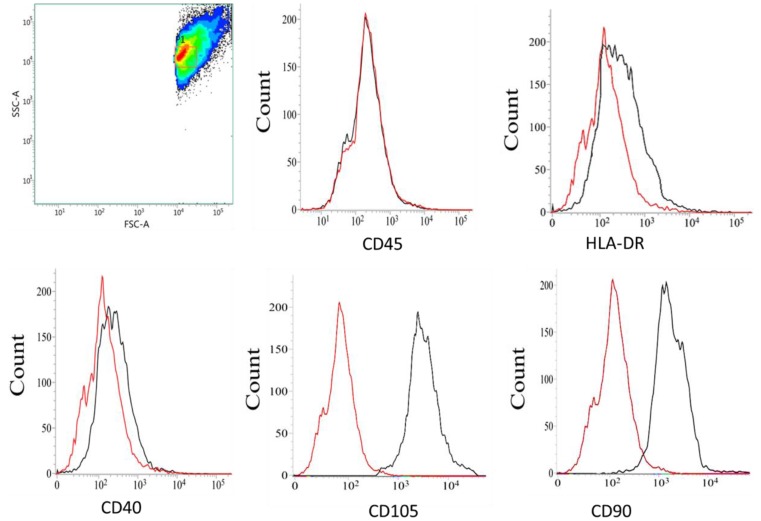
Immunophenotyping characterization of mesenchymal stem cells (MSCs) by flow cytometric analysis. Fluorescence-activated cell sorting (FACS) results confirmed that MSCs were homogenously positive for mesenchymal cell surface markers CD105 and CD90, but negative for hematopoietic cell surface marker CD45, leucocytes cell surface marker HLA-DR and macrophage cell surface marker CD40.

To confirm the phenotype of MSCs isolated from mouse compact bones, cell surface markers in MSCs were analyzed using a flow cytometer. The results showed that the MSCs expressed high level of mesenchymal surface markers CD105 and CD90, but did not express hematopoietic cell surface marker CD45; leucocytes cell surface marker HLA-DR and macrophage cell surface marker CD40 (Figure 1). Using Fluorescence-activated cell sorting (FACS) analysis, our results confirm that MSCs generated from mouse compact bone were characteristics of mesenchymal phenotype without any contamination from hematopoietic lineages, leucocytes and macrophages.

### 3.2. Tri-Lineage Differentiation of MSCs

**Figure 2:**
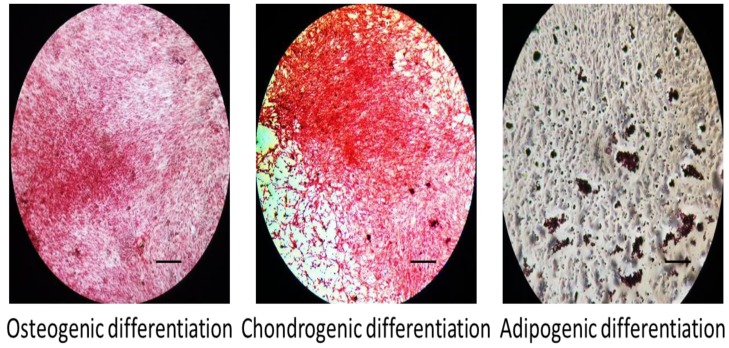
Tri-lineage differentiation potential of MSCs. Osteogenesis, chondrogenesis and adipogenesis of MSCs were evaluated by Alizarin red, Safranin-O and Oil Red-O staining, respectively, after 21 days induction. Scale bar: 1 mm.

We further investigated the tri-lineage characterization of MSCs. The tri-lineage differentiation potential of MSCs was determined by cytochemical staining with Oil Red-O (adipogenic), Alizarin red (osteoblastic) and Safranin-O (chondrogenic) (Figure 2). During osteogenic differentiation, the differentiated MSCs were positively stained for Alizarin red, confirming the presence of mineralized nodules. The positive staining of Safranin-O confirmed the presence of sulfated proteoglycon accumulation in differentiated MSCs. For adipogenic differentiation, intracellular Oil Red-O staining confirmed the deposition of lipid granules by adipose cells. From the above results, MSCs isolated from mouse compact bone possess the ability of multi-lineage differentiation.

### 3.3. Role of sRANKL and PTHrP in MSCs Proliferation

#### 3.3.1. Cell Viability

**Figure 3:**
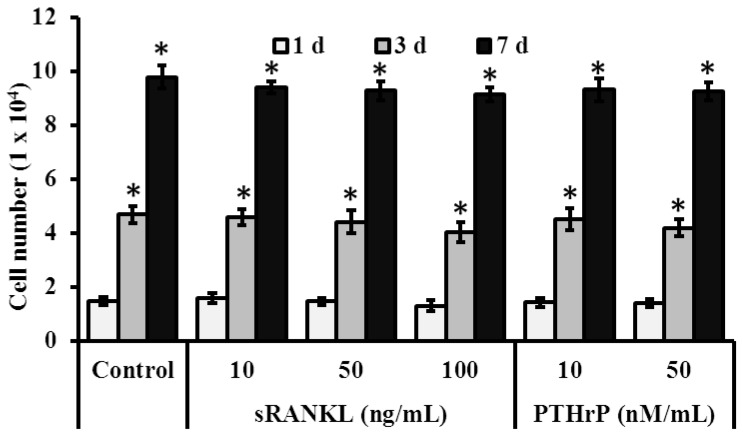
Effect of soluble receptor activator of nuclear factor-κB ligand (sRANKL) and parathyroid hormone-related peptide (PTHrP) on proliferation of MSCs. * *p* < 0.05 vs. 1 day.

Proliferation of MSCs increased with culture time (*p* < 0.05) but was not significantly affected by the treatment of sRANKL and PTHrP whatever the concentration (Figure 3).

#### 3.3.2. mRNA Expression

**Figure 4:**
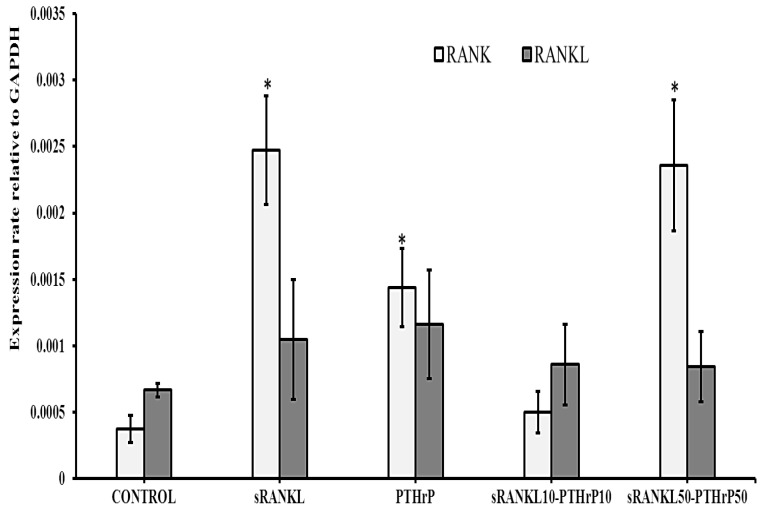
RANK and RANKL mRNA expression of MSCs treated with sRANKL and PTHrP, * *p* < 0.05 vs. control.

Since there was no difference in cell numbers, we investigated whether cellular RANK and RANKL level of MSCs were regulated by sRANKL and PTHrP treatment. Interestingly, the level of RANK was significantly upregulated by sRANKL (50 ng/mL), PTHrP (50 nM/mL) and sRANKL50–PTHrP50 compared to control (*p* < 0.05). In contrast, sRANKL10–PTHrP10 did not significantly modify RANK expression (Figure 4). In addition, RANKL mRNA levels were not affected by sRANKL and PTHrP. This indicated that sRANKL upregulated membrane RANK levels, but not RANKL levels.

### 3.4. MSCs Express Membrane RANK

**Figure 5:**
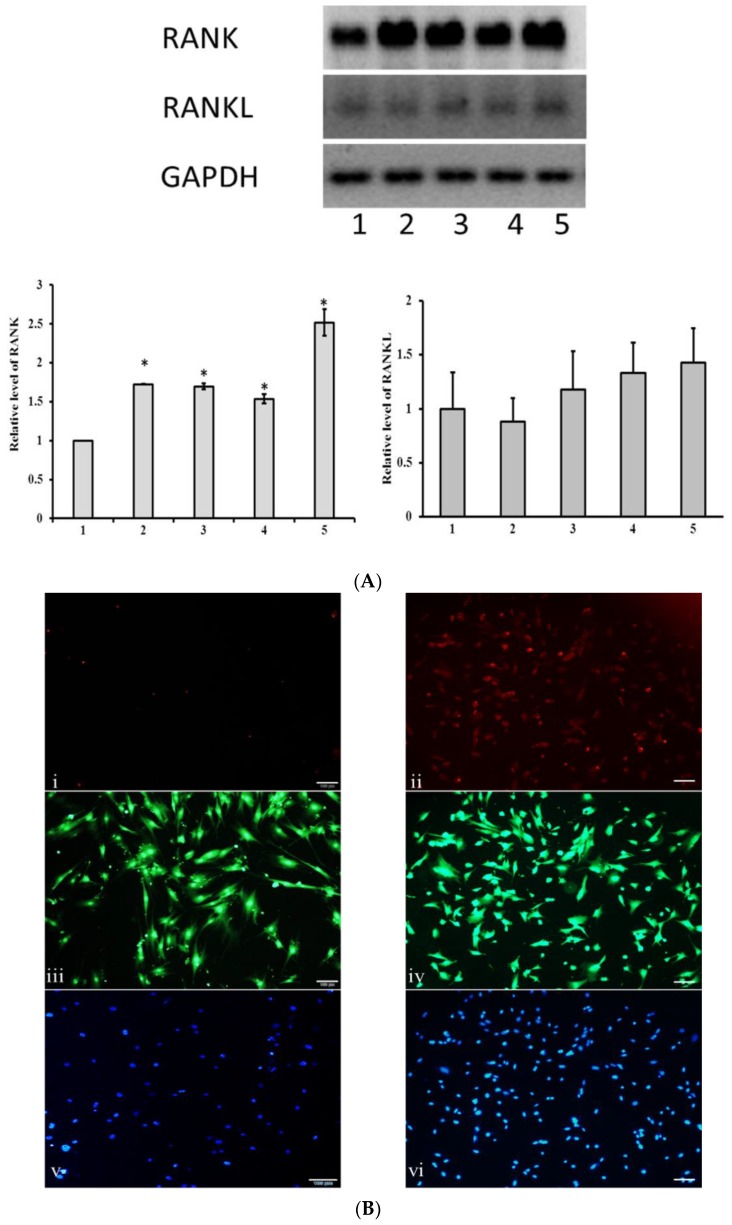
RANK expression of MSCs treated with sRANKL and PTHrP. (**A**) Western blot analysis, 1—control, 2—sRANKL50 (50 ng/mL), 3—PTHrP50 (50 nM/mL), 4—sRANKL10–PTHrP10 (10 ng/mL + 10 nM/mL), 5—sRANKL50–PTHrP50 (50 ng/mL + 50 nM), * *p* < 0.05 vs. control. (**B**) Immunocytochemistry, (**i**,**ii**), (**iii**,**iv**) and (**v**,**vi**): RANK expression, fluorescein isothiocyanate (FITC) and 4′,6-diamidino-2-phenylindole (DAPI) staining of control and sRANKL treated cells, respectively.

We next assessed protein expression of RANK and RANKL in MSCs using Western blot. The data confirmed the higher expression of mRANK in RANKL and PTHrP treated MSCs than in control MSCs (*p* < 0.05) (Figure 5A). Further, sRANKL treated MSCs had elevated expression of RANK, indicating a stimulating effect of sRANKL on mRANK in MSCs. In contrast, the level of mRANKL protein expression had no significant effect in control and treated MSCs. Based on the mRNA and Western blot experiments, we further investigated the level of cellular mRANK expression in MSCs treated with sRANKL (50 ng/mL) using immunocytochemistry. As we expected, MSCs expressed mRANK after 7-day culture when they were treated with sRANKL (50 ng/mL), but not in control cells (cells cultured without sRANKL) (Figure 5B). These results revealed that MSCs recruit sRANKL for the activation of mRANK.

### 3.5. sRANKL Downregulates Osteogenic Differentiation of MSCs

The above experiments confirmed that sRANKL upregulates mRANK expression in MSCs, however, the role of sRANKL, RANK and PTHrP in osteogenesis is not well understood. Therefore, further experiments were carried out to identify the role of sRANKL in osteogenic differentiation and to investigate the autocrine cues of MSCs in osteogenesis. Also, we investigated the role of PTHrP on sRANKL’s action in osteogenesis. For this purpose, MSCs were differentiated with osteogenic medium in the presence and absence of sRANKL and PTHrP for 21 days and measured mRNA expression (osteocalcin and collagen), protein expression (RANK, RANKL, Col_1_α_2_, osteocalcin and progressive ankylosis protein (ANK)), mineral deposition and alkaline phosphatase level.

**Figure 6:**
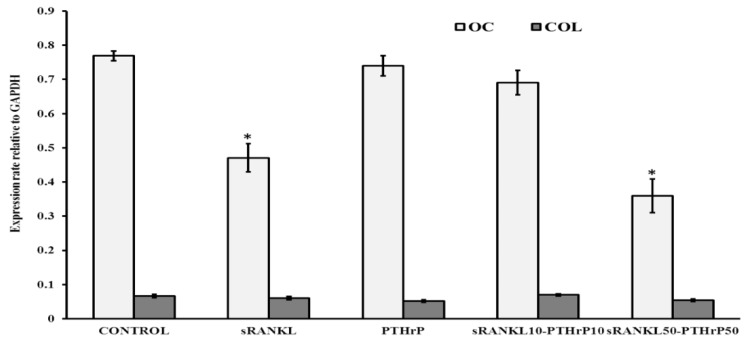
mRNA expression of differentiated MSCs by sRANKL and PTHrP. * *p* < 0.05 vs. control.

The mRNA expression of differentiated MSCs for osteocalcin was significantly downregulated by sRANKL and sRANKL50–PTHrP50 (*p* > 0.05) but not by PTHrP and sRANKL10–PTHrP10 (Figure 6). In contrast, the collagen mRNA expression was not altered by sRANKL but was slightly decreased by PTHrP50.

**Figure 7:**
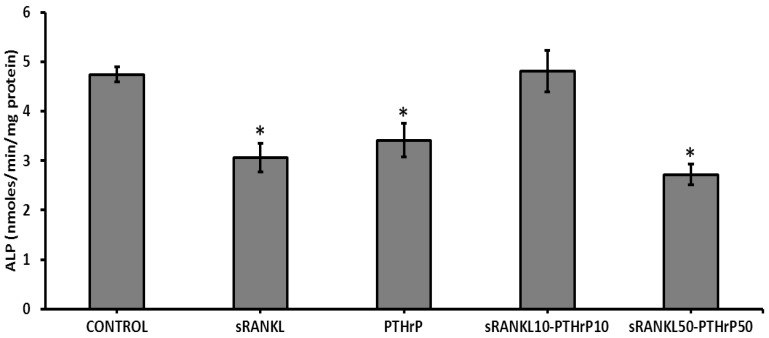
Cellular alkaline phosphatase (cALP) expression of differentiated MSCs by sRANKL and PTHrP, * *p* < 0.05 vs. control.

Cellular alkaline phosphatase (cALP) level of osteoblast is believed to be a potential hallmark biomarker of osteogenesis. Therefore, we quantified the cALP level in sRANKL treated differentiated MSCs. sRANKL diminished cALP level in the presence of osteogenic supplements compared to control (Figure 7). The lower concentration of sRANKL10-PTHrP10 had no effect. However, sRANKL (50 ng/mL), PTHrP (50 nM/mL), and sRANKL50–PTHrP50 decreased the level of cALP. This indicates PTHrP supports sRANKL-dependent inhibitory effects in osteogenesis.

**Figure 8:**
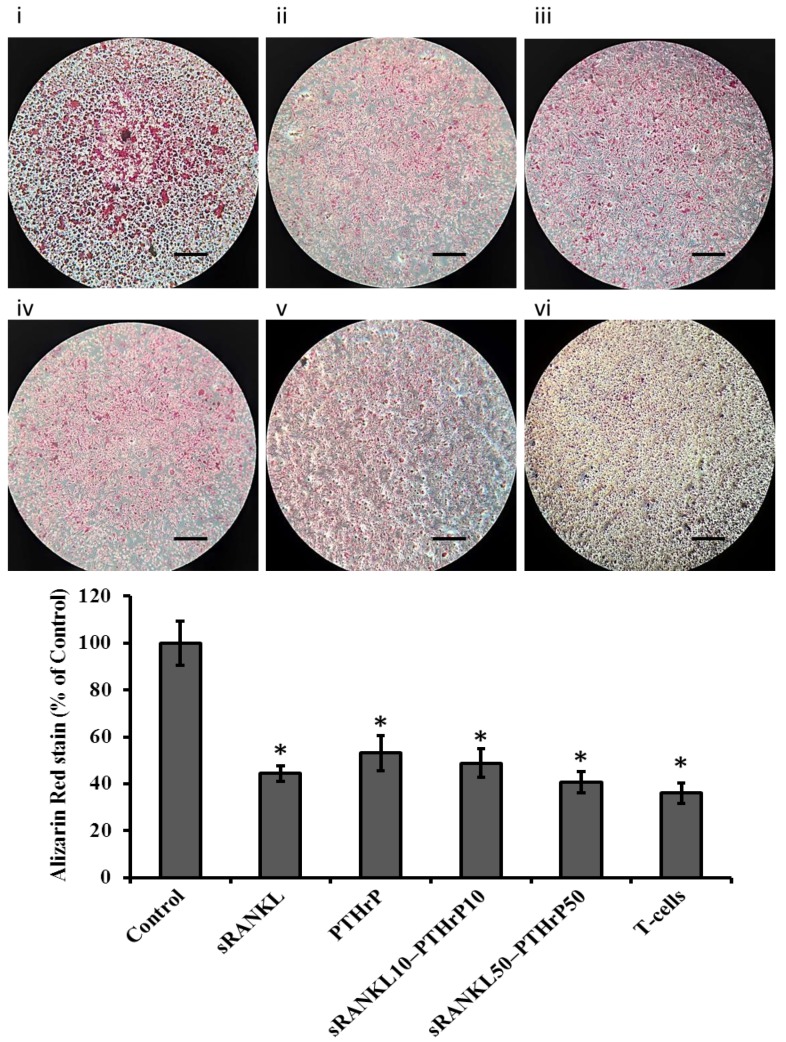
The level of Alizarin red staining of differentiated MSCs by sRANKL and PTHrP. (**i**) control, (**ii**) sRANKL, (**iii**) PTHrP10, (**iv**) sRANKL10–PTH10, (**v**) sRANKL50–PTHrP50, (**vi**) T-cells-MSCs, scale bars: 0.1 cm. * *p* < 0.05 vs. control.

**Figure 9:**
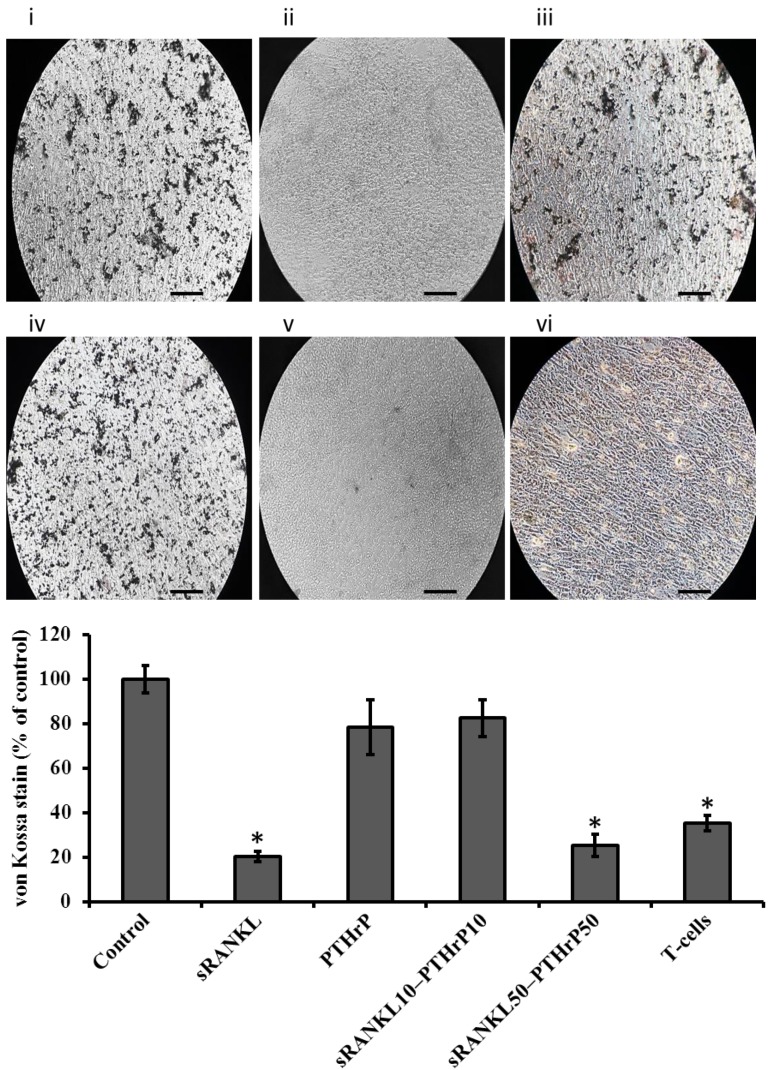
The level of von Kossa staining of differentiated MSCs by sRANKL and PTHrP. (**i**) control, (**ii**) sRANKL, (**iii**) PTHrP10, (**iv**) sRANKL10–PTH10, (**v**) sRANKL50–PTHrP50, (**vi**) T-cells-MSCs, scale bars: 0.1 cm. * *p* < 0.05 vs. control.

The effect of sRANKL and PTHrP on cellular mineral deposition of differentiated MSCs was assessed using Alizarin red staining and the von Kossa method. As expected, the level of mineral deposition was less in sRANKL and PTHrP treated MSCs than control MSCs (Figure 8 and Figure 9). However, the amount of mineralized area in sRANKL and PTHrP treated MSCs was inconsistent between Alizarin and von Kossa staining.

**Figure 10:**
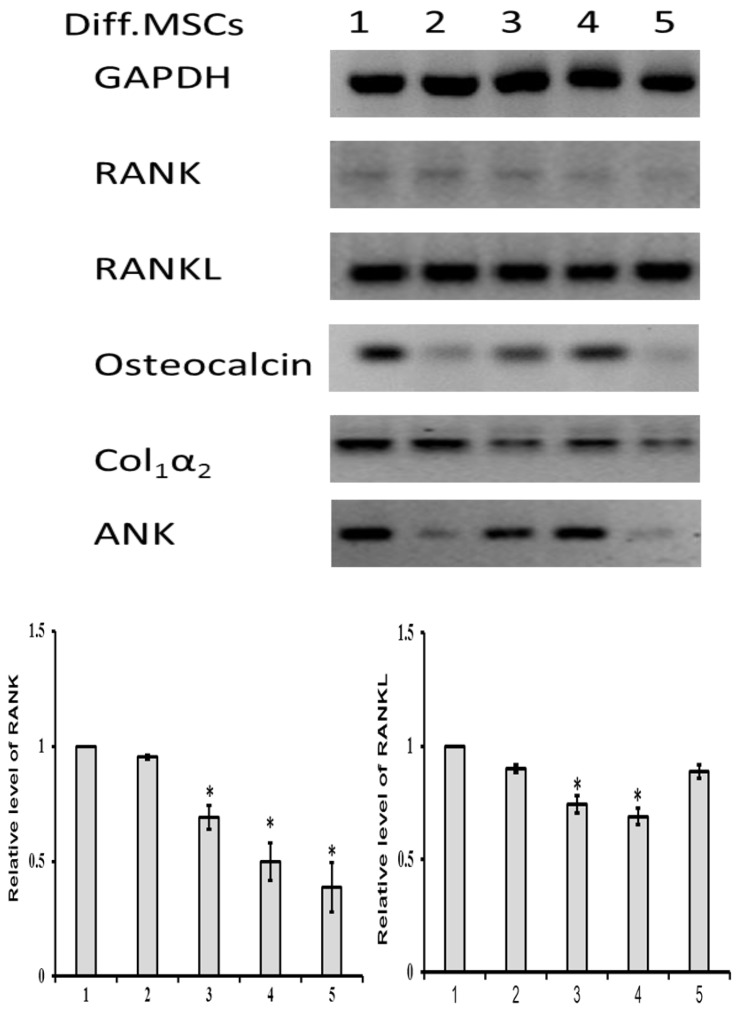

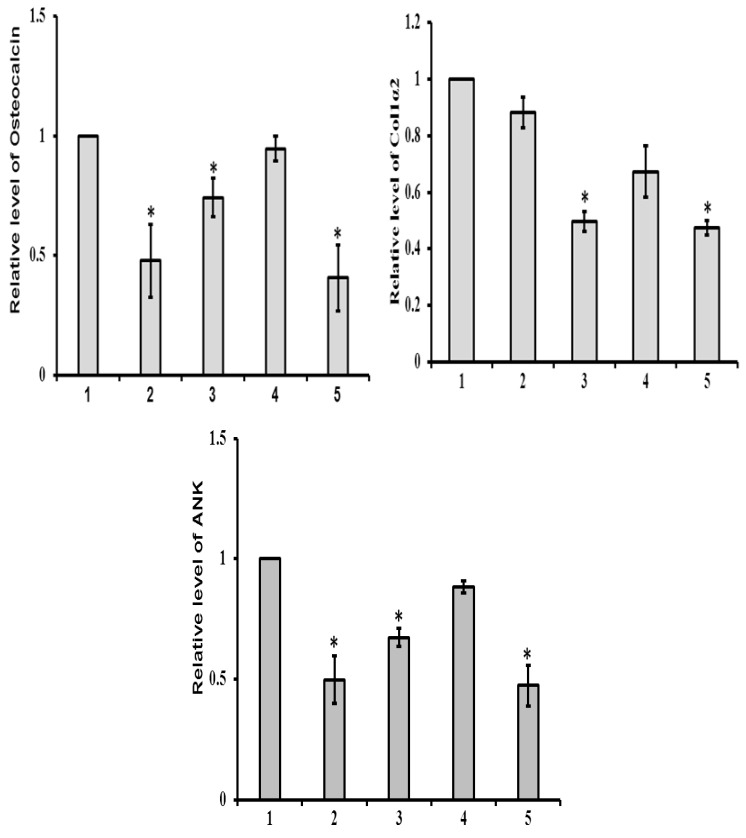
Protein expression of differentiated MSCs treated with sRANKL and PTHrP. 1—control, 2—sRANKL50, 3—PTHrP50, 4—sRANKL10–PTHrP10, 5—sRANKL50–PTHrP50. * *p* < 0.05 vs. control.

To investigate the protein expression of differentiated MSCs, Western blot analysis was carried out. As shown in Figure 10, the level of RANK expression was suppressed in sRANKL and PTHrP treated differentiated MSCs, compared to control MSCs. In contrast, the differentiated MSCs expressed high level of RANKL both in treated and control groups. The expression of RANKL was similar in all the treated groups, except PTHrP50 and sRANKL10–PTHrP10 treated MSCs.

The expression of osteocalcin protein was downregulated in sRANKL and PTHrP treated MSCs compared to the controls (Figure 10). Interestingly, the level of osteocalcin expression was significantly decreased in sRANKL50–PTHrP50 compared to sRANKL50 alone and PTHrP50 alone, indicating that the downregulating effect of osteocalcin by sRANKL was more pronounced in the presence of PTHrP.

Col_1_α_2_ protein expression was downregulated in PTHrP50 and sRANKL50–PTHrP50 treated MSCs while no changes observed in sRANKL treated MSCs.

Since the sRANKL downregulates mineral deposition of differentiated MSCs, we further investigated the signaling pathway of ANK, a major regulatory protein for mineralization. sRANKL50, PTHrP50 and sRANKL50–PTHrP50 treated differentiated MSCs had lower level of ANK protein expression compared to control MSCs (*p* < 0.05). The differentiated MSCs treated with sRANKL10–PTHrP10 showed similar protein expression of ANK compared to control MSCs. This result confirmed that sRANKL downregulates osteogenic differentiation of MSCs by decreasing mineral deposition through the inhibition of the ANK signaling pathway and this effect was more pronounced in the presence of PTHrP.

### 3.6. The Level of Secretome

**Figure 11:**
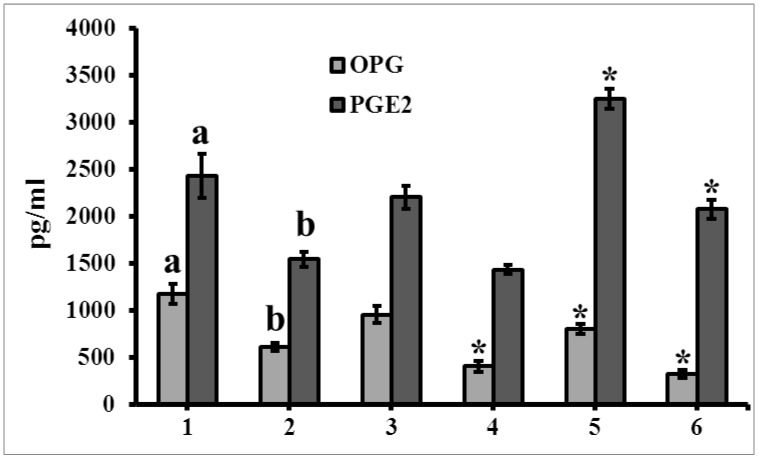
The level of secretome in MSCs treated with sRANKL and PTHrP. The osteoprotegerin (OPG) and prostaglandin E2 (PGE2) levels in MSCs cell supernatants were measured by ELISA. For osteogenic induction, MSCs were cultured with osteogenic medium, whereas for undifferentiated MSCs, cells were cultured without osteogenic medium for 21 days. 1—undifferentiated MSCs, 2—differentiated MSCs, 3—undifferentiated MSCs–sRANKL, 4—differentiated MSCs–sRANKL, 5—undifferentiated MSCs–PTHrP, 6—differentiated MSCs–PTHrP. a,b–bars with different letters, indicate statistical significance (*p* < 0.05) between undifferentiated and differentiated control MSCs. * indicates statistical significance between treated MSCs (RANKL and PTHrP) and control MSCs. *p* < 0.05.

The OPG and PGE2 levels were significantly decreased in differentiated MSCs than undifferentiated MSCs (*p* < 0.05) (Figure 11). The level of OPG was significantly decreased in both sRANKL and PTHrP treated differentiated cells compared to control differentiated cells (*p* < 0.05). In contrast, sRANKL treatment did not contribute any significant changes in PGE2 level. However, PTHrP increased the level of PGE2 in both undifferentiated and differentiated MSCs compared to control cells (*p* < 0.05).

### 3.7. T-Cells Downregulate Osteogenesis of MSCs

When they were co-culture with T-cells, MSCs showed lower level of mineral (Alizarin and von Kossa) staining compared to control (Figure 8 and Figure 9). To investigate the role of T-cells in osteogenesis further, the RANKL level was quantified in T-cells cultured for 21 day. The results showed that the level of mRANKL secreted by T-cells was 79 ng/mL, however, the sRANKL was below the detectable range by ELISA.

## 4. Discussion

Our study confirmed that the harvested MSCs from mouse compact bone displayed the characterization of mesenchymal phenotypes with no contamination from hematopoietic cells, leucocytes or macrophages. Furthermore, the tri-lineage differentiation confirmed that the compact bone MSCs can readily differentiate into osteoblasts, chondrocytes and adipocytes upon induction. It is well-known that RANK is expressed in osteoclastogenic progenitor and mature osteoclasts cell membrane in order to regulate osteoclast formation and differentiation [12,13]. A recent study identified the expression of RANK in MSCs, which play a negative role in osteogenesis of MSCs [21]. The role of RANK is always dependent on its ligand, RANKL in biological action. In this study we investigated the role of sRANKL, PTHrP and immune cells in osteogenesis of MSCs. Though MSCs proliferation was not affected by sRANKL and PTHrP, the sRANKL treatment triggered the membrane RANK expression in MSCs after 7 days and downregulated osteogenesis of MSCs. In contrast, previous work reported RANK expression in the cytoplasm of MSCs without sRANKL treatment [21]. In the present study, we identified mRANK expression in sRANKL treated MSCs, however MSCs did not express RANK in the absence of sRANKL, meaning that MSCs require RANKL for RANK activation to downregulate osteogenesis. This was confirmed by immunocytochemistry and mRNA expression of sRANKL treated MSCs.

The decrease level of cALP and mineral deposition by sRANKL was another key finding of the present study. The sRANKL effect was accelerated in the presence of PTHrP. The communication signals between PTHrP and RANKL are still unexplainable, which needs further extensive study.

MSCs express RANK at the earlier stage of osteogenesis later the cells do not express RANK at osteoblastic lineage stage. It is well known that immune cells such as lymphocytes express high level of RANKL during activation, which contributes to osteoclast formation [26,27,28]. T-lymphocytes are major source of RANKL and play crucial role in bone homeostasis [29,30]. RANKL secreted from T-lymphocytes may play the role through PKC, ERK1/ERK-2 and calcineurin regulatory signaling pathways [31,32]. It has been stated that RANKL promotes dendritic cell maturation and downregulates apoptosis for dendritic cell mediated T-cell proliferation through MHC-I class molecule [33]. RANKL produced from T-lymphocytes assists bone resorption through osteoclastogenesis. However, the induction of osteoclastogenesis by sRANKL stimulates secretion of many intercellular signaling factors from its precursors that can intermingle with neighboring cells to modulate bone size and strength. It was reported that synovial effector T-cells secreted RANKL protein from its membrane during an adjuvant-induced arthritis model [31]. 

As a major source of RANKL, we demonstrated the inhibitory role of RANKL from T-cells in osteogenesis of MSCs. For this purpose, MSCs stimulated osteogenic differentiation in the presence of T-cells as a co-culture method and the mineral deposition level of differentiated MSCs was investigated. In the present study, we also investigated whether sRANKL from T-cells conditioning-medium downregulates osteoclastogenesis. However, since sRANKL levels were as low as negligible and below the minimum detectable range by ELISA, it could not bring any changes in osteogenesis of MSCs. Therefore, a further experiment was carried out with mRANKL of T-cells through co-culture (T-cell-MSCs) to investigate the role of RANKL in osteogenesis of MSCs. Our results confirmed that mRANKL expressed in T-cells downregulated the osteogenic differentiation of MSCs.

In general, MSCs express high level of OPG, but this level was decreased during differentiation. At the same time, sRANKL and PTHrP also decreased the secretion of OPG in both undifferentiated and differentiated MSCs. Surprisingly the level of PGE2 was increased by PTHrP treatment in MSCs, which was not observed in sRANKL treated cells.

From the present study, it is confirmed that the sRANKL downregulates osteogenesis of MSCs mainly through decreasing mineral deposition of MSCs. To find the mechanism of sRANKL in decreasing mineral content, we further investigated the ANK protein expression in MSCs. From this experiment, we demonstrated that sRANKL reduces mineral deposition during osteogenesis of MSC through downregulating the ANK signaling pathway. It is well known that ANK encodes a multiple-pass transmembrane protein that regulates PPi transport from the cytoplasm to the extracellular milieu [33,34]. A mouse mutation of ANK showed severe joint mineralization and arthritis [33,35,36]. Therefore, downregulation of ANK by sRANKL may responsible for decreased mineral deposition in MSCs.

It is evidenced that RANKL from osteoblasts and osteocytes plays a major role in activating osteoclast formation from its precursor cells. Here we report the RANK and RANKL expressed in MSCs regulate paracrine and autocrine signaling for osteogenesis and osteoclastogenesis. During the earlier stage, MSCs express RANK by sRANKL stimulation, which may bind with RANKL of neighboring osteogenic cells to control excess bone formation. When MSCs differentiate to osteoblasts, the mature osteoblasts express RANKL to activate osteoclast formation for bone resorption.

## 5. Conclusions

Overall, the present in vitro results disclose the autocrine signaling mechanism of MSCs that when MSCs are differentiated into osteogenic cells, the differentiated MSCs produce a negative feed-back signal through releasing sRANKL, which can activate mRANK in MSCs, thereby downregulating osteogenic differentiation to prevent excessive bone formation. This study specifically concludes that sRANKL:

1. Did not affect MSCs proliferation;
2. Increased RANK expression in MSCs;
3. Downregulated osteogenesis of MSCs;
4. Decreased mineral deposition of differentiated MSCs;
5. Downregulated ANK protein expression;
6. Regulated by PTHrP;
7. Expression increased in differentiated MSCs;
8. mRANKL from T-cells downregulate osteogenesis.

The major limitations of this study are:

1. Although we identified the downregulating osteogenic differentiation of MSCs by sRANKL through decreasing ANK protein expression, the actual mechanism of hematopoietic stem cells, osteoclasts and immune cells in downregulating osteogenesis through RANKL need to be verified.
2. In the present in vitro study, cells were cultured in 2D culture systems, which do not mimic the actual in vivo environment, thus further in vitro studies are needed using a 3D culture system.
3. This study lacks the actual paracrine signaling mechanism of PTHrP on RANK towards osteogenesis, and more research is needed to investigate certain hypotheses such as how the PTHrP upregulates RANKL’s action through scrutinizing the major communication between them and also the need to confirm these effects using in vivo studies.

## Supplementary Materials

The following are available online at https://www.mdpi.com/2077-0383/8/6/836/s1, Figure S1: Phase contrast microscopic structure of mesenchymal stem cell harvested from BALB/c mouse compact bone.
