# Supplementary material for: Parathyroid Hormone-Related Protein (PTHrP) Accelerates Soluble RANKL Signals for Downregulation of Osteogenesis of Bone Mesenchymal Stem Cells
Source: J Clin Med. 2019 Jun 12;8(6):836. doi: 10.3390/jcm8060836 (PMC6616973; doi:10.3390/jcm8060836)
Supplement: Supplementary file 1 [file jcm-08-00836-s001.pdf]

Journal of
Clinical Medicine

MDPI

Article

Parathyroid Hormone-Related Protein (PTHrP) Accelerates Soluble RANKL Signals for Downregulation of Osteogenesis of Bone Mesenchymal Stem Cells

Jeevithan Elango [1], Saeed Ur Rahman [2], Yves Henrotin [3], José Eduardo Maté Sánchez de Val [4], Bin Bao [1], Shujun Wang [5], Bailin Li [1] and Wenhui Wu [1,6,*]

1. Department of Marine Bio-Pharmacology, College of Food Science and Technology, Shanghai Ocean University, Shanghai, 201306, China; srijeevithan@gmail.com (J.E.); bbao@shou.edu.cn (B.B.); blli@shou.edu.cn (B.L.)
2. Interdisciplinary Research Centre in Biomedical Materials (IRCBM), COMSATS University Islamabad, Lahore Campus, Punjab, 54000, Pakistan; saeedbio80@gmail.com
3. Bone and Cartilage Research Unit, Arthropôle Liège, University of Liège, CHU Sart-Tilman 4000, Liege, Belgium; yhenrotin@ulg.ac.be
4. Department of Biomaterials Engineering, Universidad Católica San Antonio de Murcia, 30107 Guadalupe, Murcia, Spain, jemate@ucam.edu
5. Co-Innovation Center of Jiangsu Marine Bio-industry Technology, Huaihai Institute of Technology, Lianyungang, 222005, China; shujunwang86@163.com
6. National R&D Branch Center for Freshwater Aquatic Products Processing Technology (Shanghai), Shanghai 201306, China

* Correspondence: whwu@shou.edu.cn; Tel.: +86 21 61900364

Received: 17 April 2019; Accepted: 7 June 2019; Published: 12 June 2019

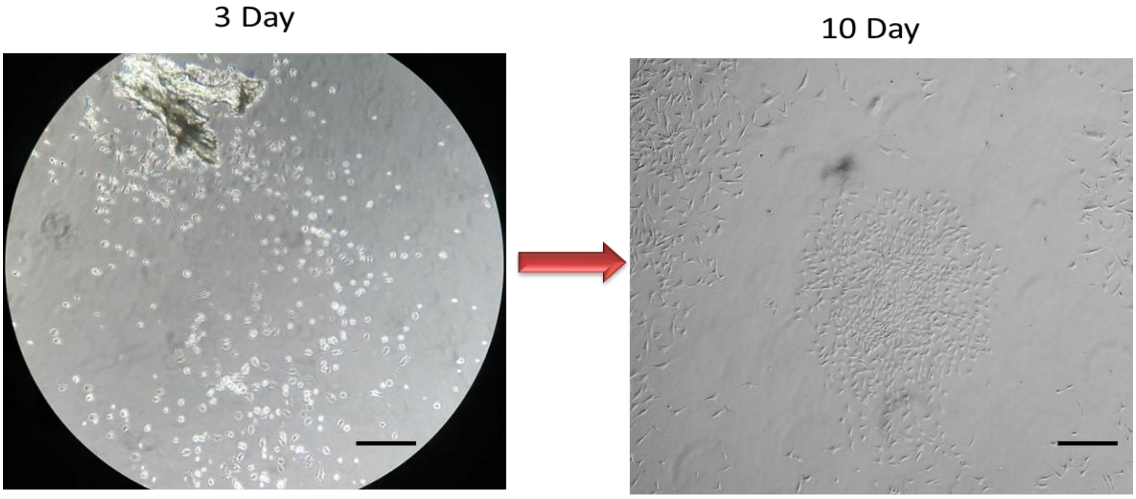

Figure S1. Phase contrast microscopic structure of mesenchymal stem cell harvested from BALB/c mouse compact bone. (**A**) 3 day culture (Bar-100 micrometer) (**B**) 10 day culture (Bar-50 micrometer).

© 2019 by the authors. Licensee MDPI, Basel, Switzerland. This article is an open access article distributed under the terms and conditions of the Creative Commons Attribution (CC BY) license (http://creativecommons.org/licenses/by/4.0/).
